# Scoliosis and the Crawl Treatment

**Published:** 1912-07-20

**Authors:** 


### THE IDEAL SCHOOL CLINIC.

### Scoliosis and the Crawl Treatment.

Home treatment, combined with class supervision, is sufficient in the slighter cases of scoliosis when it is conscientiously and persistently carried on. But in the graver degrees, especially those associated with marked elevation of the shoulder, the clinic must come to the rescue and undertake the treatment. Arrangements must be made to treat these cases in batches. The patients from two or more schools in the neighbourhood must be joined in a "scoliosis regiment" which visits the clinic on certain days every week, where they are received by the nurse and put through the proper exercises. The member of the clinic staff whose duty it is to see after these orthopædic cases must be in attendance on such occasions and must make a point of examining each child. A record of the progress of the case must be kept, and the details of treatment ordered must be written down either by the nurse or preferably by the medical man himself. It is as well that the officer on duty should make the examinations himself and not delegate the duty to another, as in the latter case the necessary criteria are lost since individual opinion and judgment must play a large part in estimating the extent of relatively small degrees of scoliosis and the progress made in curing them.

### The Treatment of Scoliosis.

The question of what treatment must be adopted at the clinic cannot be answered generally. There is no panacea for scoliosis, and it is idle to assert that one treatment based on one line, one theory, or one method alone succeeds in curing a majority of cases. Here, perhaps, more than in any other orthopædic deformity, the treatment should be based on a study of the child's individuality. Some children get little benefit from passive exercises, and improve rapidly when allowed to do active exercises: in others the reverse is the case, and apparatus work holds out the best chance of speedy success. No hard-and-fast rule can be laid down, but the nearest generalisation one can make is that in most cases a combination of active and passive movements is found to be the best.

One point it is well to lay stress on, and that is the urgent necessity of treating accessory and subsidiary lesions, whether they are the patent cause of the scoliosis or not. Where the child is anæmic and pale, a course of iron is indicated: the compound syrup of iron is, perhaps, the best tonic that can be given. Organic iron preparations are much more expensive and not a whit superior. Indigestion demands treatment, and headaches and lassitude raise a suspicion of eye strain which should be investigated as an indispensable preliminary. In fact, an examination of the eyes should be included in the routine inspection of children suffering from lateral curvature.

A room in the clinic should be set apart for the treatment of the scoliosis. For this reason it is preferable to have one or two special mornings or afternoons during the week, since the waiting-room is generally the largest and most convenient. If the crawl method is included in the treatment methods at the clinic (and in our opinion it is one of the best and most successful of the many methods now practised), care must be taken that the floor is smooth and free from splinters. A strip of linoleum is easily laid down and serves admirably for a crawl line. Crawling is, of course, best done in the open, but in this country it would be difficult always to secure an adequate crawl space in the open air; and, therefore, the method must be practised in the waiting-hall, care being taken that free ventilation is secured. At the same time the hall must be kept warm in winter, for, though crawling is a heating exercise, the children waiting their turn are apt to feel the cold when the room is not properly warmed.

### The Crawl Treatment Abroad.

At Berlin the crawl treatment is undertaken at the Royal Surgical Clinic by specially trained nurses who deal with batches of thirty children at a time. The results obtained so far are most striking and show that the method merits general adoption. To obtain the best results the crawl treatment must be combined with gymnastic and passive and active movements on the bench or Swedish ladder. The use of complicated and expensive apparatus, such as are required for the Zander and Schultess methods, is unnecessary at the clinic. Cases severe enough to require such apparatus should be sent to a proper orthopædic hospital. Massage and passive movements are useful auxiliaries, and the nurse in charge of the squad should be proficient in both. At first it is desirable to limit the "scoliosis regiments" to half a dozen children. When the nurse and the medical man get experience in the application of the methods, the number may be increased to a dozen. A larger number than this is not desirable since one supervisor cannot give adequate attention to every child in the squad when there are more than a dozen children. Fifteen children per nurse is the number allowed at Berlin, but there the nurses have had a very extensive experience, and the exercises are carried out at what is one of the best orthopædic institutes in the world, and every case has a chance of almost daily inspection.

When the scoliosis has been cured or so much improved that the child can be dismissed from the clinic, care must be taken that a bad posture is not again adopted. It is as well, therefore, that the clinic should keep in touch with old patients, and that an annual or six-monthly inspection of those dismissed from the clinic squads should be held by the doctor or nurse. Those found relapsing should then again be induced to submit to treatment. Relapses are far more common than is supposed, and without this safeguard the work of the clinic is likely to be less useful to the schools than it should be.

